# Transparent and Flexible Mayan-Pyramid-based Pressure Sensor using Facile-Transferred Indium tin Oxide for Bimodal Sensor Applications

**DOI:** 10.1038/s41598-019-50247-4

**Published:** 2019-10-01

**Authors:** Minhyun Jung, Sujaya Kumar Vishwanath, Jihoon Kim, Dae-Kwan Ko, Myung-Jin Park, Soo-Chul Lim, Sanghun Jeon

**Affiliations:** 1 Korea Advanced Institute of Science and Technology (KAIST), School of Electrical Engineering, Daejeon, 34141 Republic of Korea; 2 Division of Advanced Materials Engineering, Kongju National University, Cheonan, Chungchungnam-do 331-717, Republic of Korea; 3 Department of Mechanical, Robotics and Energy Engineering, Dongguk University, Seoul, 04620 Republic of Korea

**Keywords:** Electrical and electronic engineering, Electronic devices

## Abstract

Transparent and conducting flexible electrodes have been successfully developed over the last few decades due to their potential applications in optoelectronics. However, recent developments in smart electronics, such as a direct human-machine interface, health-monitoring devices, motion-tracking sensors, and artificially electronic skin also require materials with multifunctional properties such as transparency, flexibility and good portability. In such devices, there remains room to develop transparent and flexible devices such as pressure sensors or temperature sensors. Herein, we demonstrate a fully transparent and flexible bimodal sensor using indium tin oxide (ITO), which is embedded in a plastic substrate. For the proposed pressure sensor, the embedded ITO is detached from its Mayan-pyramid-structured silicon mold by an environmentally friendly method which utilizes water-soluble sacrificial layers. The Mayan-pyramid-based pressure sensor is capable of six different pressure sensations with excellent sensitivity in the range of 100 Pa-10 kPa, high endurance of 10^5^ cycles, and good pulse detection and tactile sensing data processing capabilities through machine learning (ML) algorithms for different surface textures. A 5 × 5-pixel pressure-temperature-based bimodal sensor array with a zigzag-shaped ITO temperature sensor on top of it is also demonstrated without a noticeable interface effect. This work demonstrates the potential to develop transparent bimodal sensors that can be employed for electronic skin (E-skin) applications.

## Introduction

One form of new electronic technology to monitor human health in the form of smart robots or humanoids with a range of different electronic sensors is known as electronic skin, or E-skin. Artificial skin is being developed to mimic the functionalities of human skin. Similar to human skin, artificial skin is typically integrated with various sensors, such as pressure, humidity, tactile, and temperature sensors. Moreover, these sensors should be flexible and soft^1–6^. However, pressure and temperature sensors, the key components in electronic skin, have been continuously developed and currently have large areas and high sensitivity levels^7–12^. To utilize pressure sensors, many different materials and architectures are used with a range of devices schemes. These include conductive nanostructures (e.g., nanoparticles, carbon nanotubes, and nanosheets), conducting polymers, and graphene mixed with polymeric materials^13–16^. Pan *et al*. used a hollow-sphere microstructured resistive pressure sensor with conductive polymer films and achieved the highest sensitivity of 137 K Pa^−1^ ^13^. Tian *et al*. showed wide working range from 0 to nearly 50 kPa with laser-scribed flexible graphene pressure sensors^14^, and Lee *et al*. demonstrated feasible tunable sensitivity using different microstructured rubber dielectric layers^15^. Similarly, high-performance and flexible temperature sensors for E-skin consisting of different materials were demonstrated^11,12,16^.

For practical E-skin, integration with multiple sensors with different sensing mechanisms into a single chip must be realized. Few attempts have been made to utilize bimodal or multimodal sensors^17–20^. In our previous reports, we showed that assimilating independent sensors into a single pixel is a convenient tactic by which to assess a bimodal sensor^7–9^. However, doing so requires different material systems to fabricate the different sensors. For example, in one study^7^, we used multiwall carbon nanotubes (MWCNTs) for a pressure sensor and PEDOT:PSS and silver nanoparticles (AgNP) for temperature sensors. To avoid the need to prepare multiple materials separately, a suitable material is required for a transparent multi-pixel bimodal sensor.

However, less interest has been shown with regard to transparent and conducting metal oxides in flexible bimodal sensors due to their requirement of high-temperature processing and their rigidness^21,22^. Despite the rigidness of crystalline ITO, it has several advantages, such as its use of a commercially established process for large-area applications, high conductivity, and high transparency^23–25^. However, the lack of flexibility of ITO is not fully utilized in all applications, especial in flexible electronic applications. Instead, these applications use alternative materials such as conductive polymers, nanowires (metal oxide, Ag and Cu), and multilayer graphene^26–29^. However, the long-term stability of these materials remains unknown. To overcome the lack of flexibility of inorganic thin metal oxides and ITO, few reports have suggested embedding these inorganic metal oxide materials into soft plastic substrates^30–32^. For example, Kang *et al*. demonstrated polycrystalline ITO embedded into a transparent polyimide substrate using silver thin film as a sacrificial layer^30^. Yang *et al*. embedded a hybrid structure of Ag/ITO from copper sacrificial layers into soft polydimethylsiloxane (PDMS)^31^. Yao *et al*. transferred ITO/Si into PDMS by means of a type of transfer technology^32^. However, in these cases, harmful chemical etchants were used to transfer the required inorganic thin films. An obvious improvement would be an environmentally friendly method to transfer all types of inorganic metal oxide materials onto plastic substrates.

In the present work, ITO is detached from a silicon substrate using an environmentally friendly method which utilizes water-soluble sacrificial layers. In this case, ITO is deposited by radio-frequency sputtering (RF sputtering) onto water-soluble sacrificial layers and then separated from this substrate using hot water. Using this simple technique, we fabricated Mayan-pyramid-shaped ITO which was embedded into PDMS as a piezo-resistive pressure sensor. These pressure sensors show an excellent working range of pressure detection (100 Pa to 10kPa), high endurance of 10000 cycles, and rapid response and recovery times of approximately 120 ms and 80 ms, respectively. To realize a bimodal sensor, we employed a temperature sensor vertically on top of the Mayan-pyramid pressure sensor, which also allows the bimodal sensor to be transparent and flexible. As noted in our earlier reports, with this approach, an external signal is transduced into distinct electric signs, and the noise is reduced upon data decoupling when dissimilar physical stimulations are applied at one time. To realize a thermal sensor, we prepared zigzag-patterned ITO using a photolithography technique. It showed sensitivity of −9.08% per degree (°C) with a thermal change of 5 °C. Additionally, we demonstrated a 5 × 5 pixel transparent and flexible bimodal sensor array by applying both pressure and temperature simultaneously in real time.

## Results and Discussion

### Transparent triple layer pyramid pressure sensor

**Figure 1:**
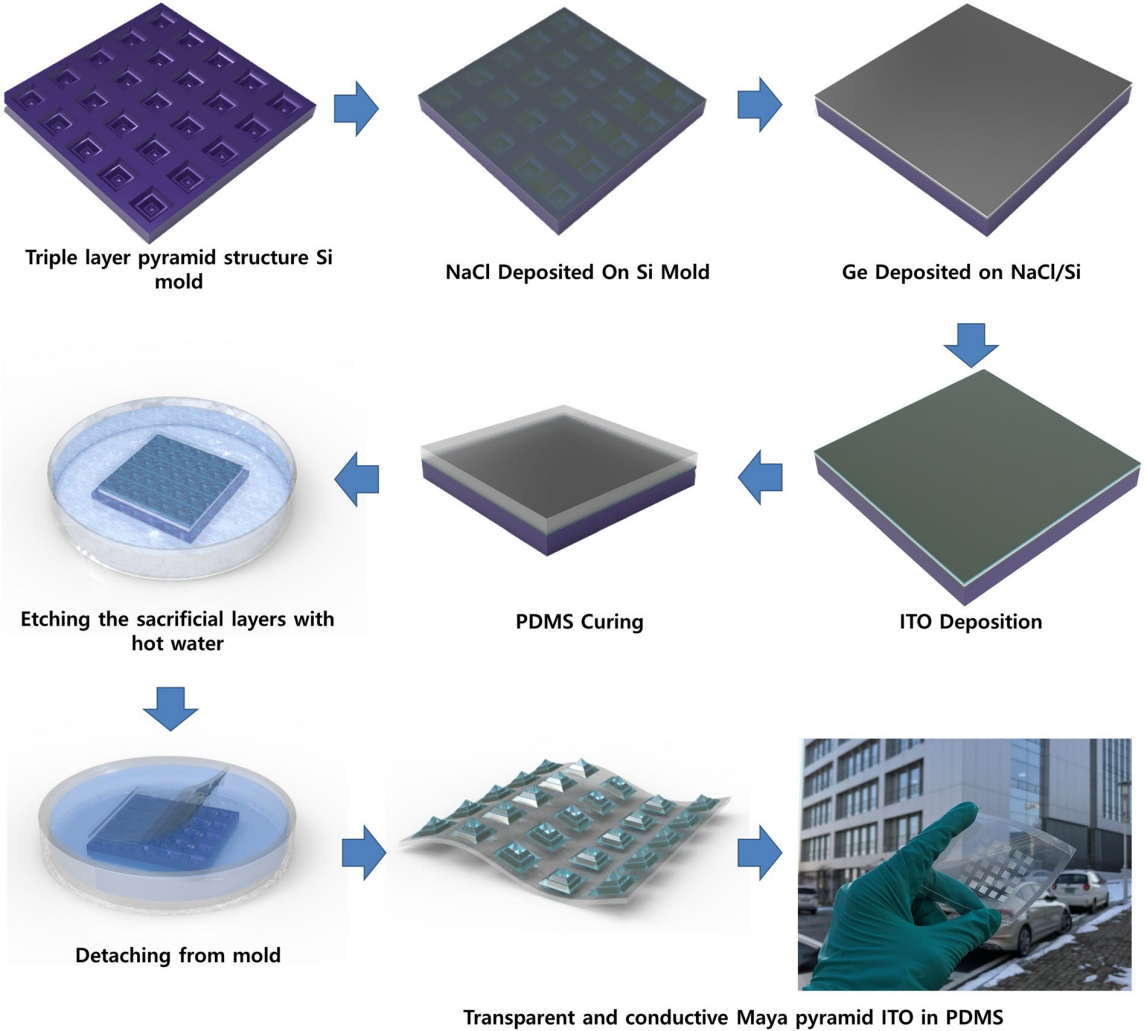
Schematic of the fabrication process of the transparent conductive substrate for a pressure sensor, and schematic images showing each step when transferring the ITO onto the flexible PDMS substrate.

In many reports, microstructure pressure sensors are fabricated by the direct deposition of conducting materials onto a PDMS mold^7–9,33,34^, possibly leading to an adhesion problem between the conducting material and the PDMS. In the present work, we embedded ITO into PDMS. The ITO acts as a bottom electrode for the pressure sensor. Figure 1 shows a schematic view of the fabrication process used to create the transparent Mayan-pyramidal pressure sensor.

Water-soluble sacrificial layers (sodium chloride (NaCl): 200 nm and germanium (Ge): 50 nm) are deposited using a thermal evaporator under a vacuum onto a patterned silicon mold. Subsequently, 250-nm-thick tin-doped indium oxide (ITO) is deposited onto these layers by means of RF sputtering. We transferred the ITO onto polydimethylsiloxane (PDMS) by dissolving the sacrificial layers in hot water. Both NaCl and Ge dissolved less than 15 mins in water at a temperature of 70 °C. Finally, the flexible, transparent and conducting triple layer on the Mayan-pyramid-structured ITO is transferred from the rigid silicon mold. This flexible and patterned ITO showed transparency which exceeded 80% (as shown in Fig. S1 in the Supporting Information) and sheet resistance up to 2 kΩ/ϒ. We confirmed that there were negligible amounts of Ge and/or NaCl residue on the embedded flexible ITO substrate via energy dispersive X-ray spectroscopy measurements, as shown in Fig. S2 (Supporting Information). The flexible integrity of the film with the ITO embedded into the PDMS substrate compared to ITO film directly deposited onto PDMS was estimated by outer and inner bending and then by measuring the relative resistance change $(R/Ro)$, where R is the resistance change after bending and Ro is the initial resistance.

**Figure 2:**
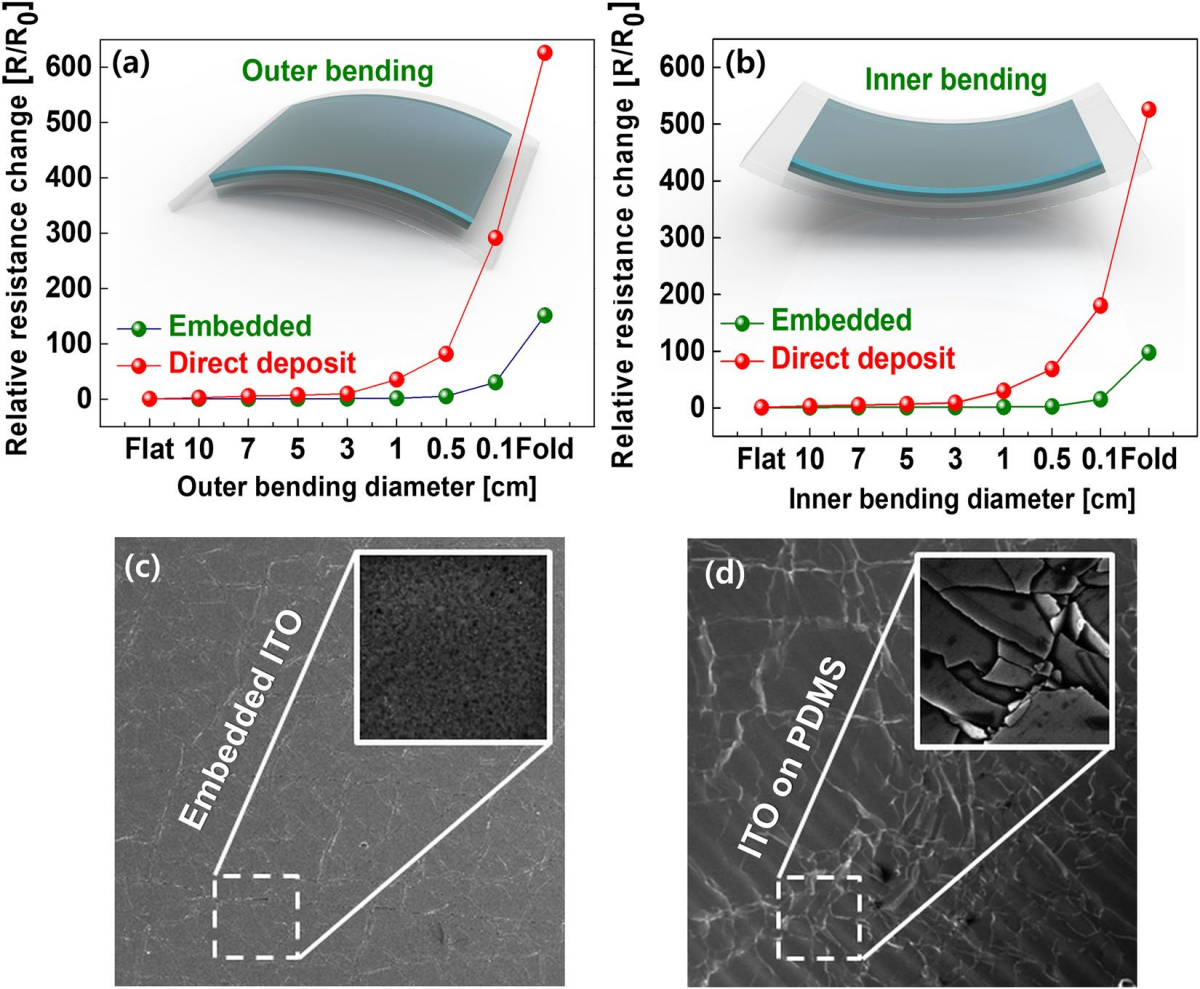
Mechanical integrity of the 200 nm ITO film embedded into a PDMS substrate: (**a**) outer bending and (**b**) inner bending test compared to 200 nm of ITO film deposited on to PDMS. SEM images of Flexible ITO embedded in PDMS (**c**) and deposited on PDMS.

As shown in Fig. 2a,b, the films with embedded ITO were found to be more stable than those with directly deposited ITO according to both the outer and inner bending measurements because less strain is induced on the ITO film when it is embedded into the soft PDMS substrate rather than being directly deposited^30,35,36^. In both films, cracks were observed after bending the films by more than 0.5 cm, as shown in Fig. 2c,d. However, the films with embedded ITO showed fewer cracks, implying that these films in PDMS can be utilized for pressure sensor applications.

**Figure 3:**
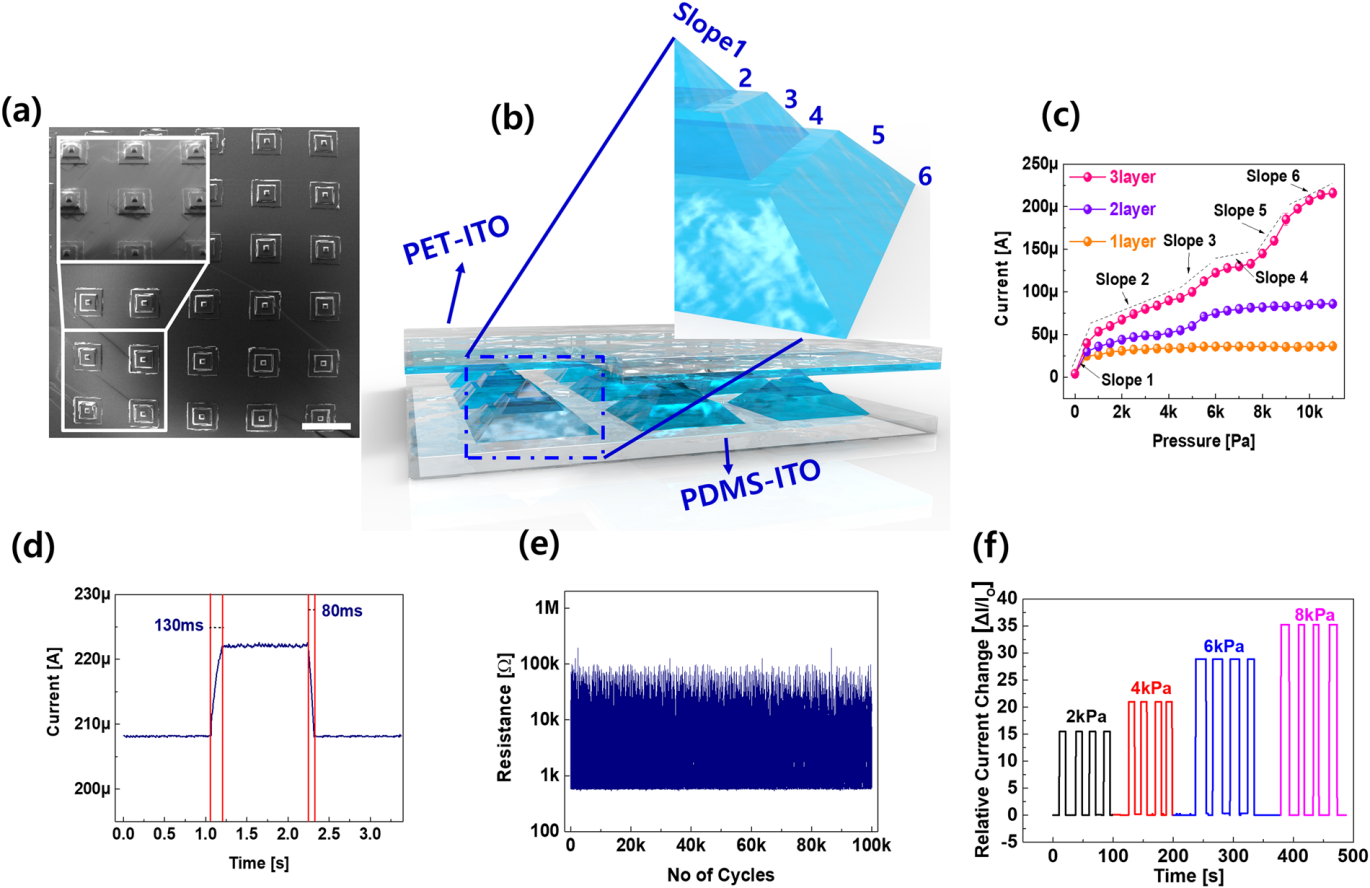
Overview of the flexible and transparent Maya pyramid pressure sensor: (**a**) SEM image of the Maya pyramid structure ITO. Inset shows a zoomed-in image. The scale bar is 1 mm. (**b**) Schematic image of each Maya pyramid structure pressure sensor. (**c**) Electrical characteristics of the pressure sensor as a function of the applied pressure with various layer structures. (**d**) Response times of the pressure sensor. (**e**) Endurance test results of the pressure sensor under 1k Pa. (**f**) Reproducibility of the electrical properties at various pressures.

Figure 3a depicts the scanning electron microscope (SEM) microstructure of the Mayan-pyramid ITO embedded into the PDMS substrate, which acts as a piezoresistive electrode with ITO-coated PET used as a counter electrode in a pressure sensor. Generally, a piezoresistive sensor works on the principle of a change in the resistance caused by an applied force. In other words, when force is applied to the counter electrode or top electrode of the pressure sensor, the resistance changes with respect to the bottom electrode due to the deformation of the soft PDMS^37–39^. However, the architecture of the piezoresistive electrode plays an important role in the pressure sensing response^33,40,41^. The Mayan-pyramid ITO bottom electrode can access six different types of slope responses, as shown in Fig. 3b,c. Slope 1 corresponds to the first contact between the counter electrode and the tip of the Mayan-pyramid pressure sensor; slope 1 is sharp due to the change in the contact area. At slope 2, the counter electrode is in contact with the base of the first layer of the Mayan pyramid and shows a slight saturation current; however, we noted that the current increased at slope 3 because the counter electrode comes into contact with the tip of the second layer of the Mayan pyramid. Later we observed similar responses from slopes 4, 5 and 6. In addition, we compared the current responses from pressure sensors with single- and double-pyramid structures, as shown in Fig. 3c. The sensing mechanism of the triple-layer or Mayan-pyramid structure pressure sensor functions depending on changes of the current induced by structural stress. At slope 1, the change in the current at the pressure sensor can be simply explained by Ohm’s law, (as shown in Eq. (1)) with the operation voltage (V), the contact area between the top and bottom electrode (A), the resistivity (ρ), and the compressed thickness of the pyramid structure (d).

1 $$\varDelta {\rm{I}}\propto \frac{VA}{\rho }(\frac{1}{d-{d}_{c}}-\frac{1}{d})$$

Note that $\frac{{d}_{c}}{d}=\varepsilon$ is the compressive strain; hence, Eq. (1) can be rewritten as

2 $$\varDelta {\rm{I}}=\frac{VA}{\rho d(\frac{1}{\varepsilon }-1)}.$$

Considering the applied compressive stress (δ) and elastic modulus (E), Eq. (2) can be written as

3 $$\varDelta {\rm{I}}=\frac{VA}{\rho d(\frac{E}{\delta }-1)}.$$

Therefore, if the operation voltage and resistivity of the coated material are constant, the change in the current shows inverse dependency on the compressive stress and elastic modulus, leading to a change in the thickness of the PDMS.

The response time is another key factor when evaluating a pressure sensor when low pressure is applied. In this case, the Mayan-pyramid-structured sensor showed rapid responses of the rise and fall times of approximately 130 ms and 80 ms, respectively, as shown in Fig. 3d. In addition, 10^5^ cycles of stable loading and unloading cycles were observed under 1k Pa of force, as shown in Fig. 3e, which provides evidence of the excellent reproducibility of pressure sensing in the Mayan-pyramid pressure sensor. Moreover, we confirmed the relative current changes at different applied pressures ranging from 2 K Pa to 8 K Pa, as shown in Fig. 3f, suggesting the feasibility of the device under a wide range of pressures.

### Pressure sensor applications

**Figure 4:**
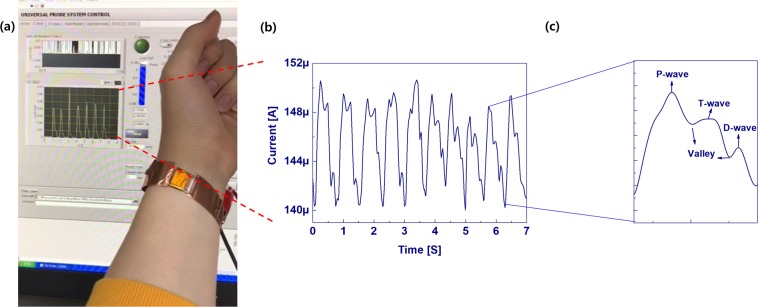
Electrical characteristics of the Maya pyramid pressure sensor: (**a**) image of the measurement setup, (**b**) data from the blood pressure monitoring assessment using the pressure sensor, and (**c**) a replot of a single pulse.

Human health nursing is one area where pressure sensors can be applied. Here, we demonstrated real-time wrist pulse measurements to provide cardiovascular monitoring in cases such as atherosclerosis and hypertension. To do this, the sensor is attached to the wrist with the help of polyimide (PI) tape, as shown in Fig. 4a. The corresponding wave forms are replotted in Fig. 4b,c. The obtained pulse rate is about 71 ~72 beats/min. The replotted regular wrist pulse wave represents the blood pressure in the form of a P-wave (percussion) and a T-wave (tidal). The D-wave (diastolic or dicrotic) denotes the heart beat and the valleys^42,43^, as shown in Fig. 4c.

### Pressure sensor applications

**Figure 5:**
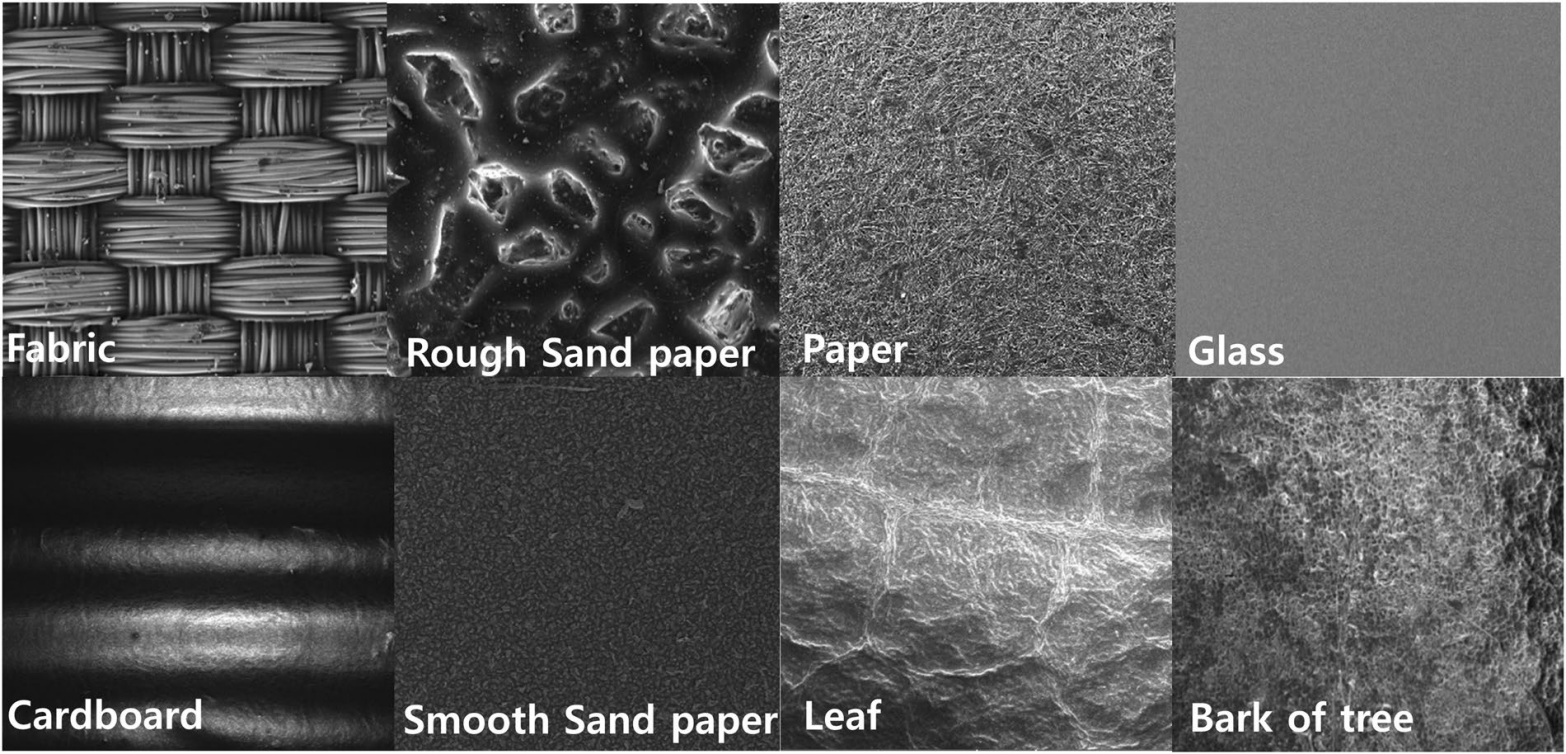
Surface SEM images of different textures for tactile sensing.

**Figure 6:**
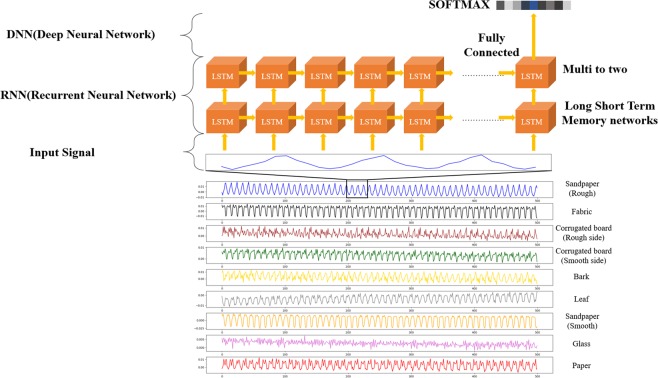
Texture Classification architecture using LSTM and FC layered of neural network from electric signal during rubbing on the texture with the triple layer pyramid structure pressure sensor.

Human health nursing is one area where pressure sensors can be applied. Here, we demonstrated real-time wrist pulse measurements to provide cardiovascular monitoring in cases such as atherosclerosis and hypertension. To do this, the sensor is attached to the wrist with the help of polyimide (PI) tape, as shown in Fig. 4a. The corresponding wave forms are replotted in Fig. 4b,c, respectively. The obtained pulse rate is about 71 ~72 beats/min. The replotted regular wrist pulse wave represents the blood pressure in the form of a P-wave (percussion) and a T-wave (tidal). The D-wave (diastolic or dicrotic) denotes the heart beat and the valleys^42,43^, as shown in Fig. 4c. Identification of the material properties is one of the most important cues that a robot has successfully interacted with its surroundings or with a human^44^. Strain gauges and force sensors or tactile sensors are used to detect vibrations during object-sensor interaction for material classification purposes. Surface recognition usually involves learning through a frequency-domain analysis of vibrations detected by an accelerometer with machine learning algorithms, such as the support vector machine (SVM) or a k-nearest neighbor (k-NN) algorithm^45^. Recently, deep learning has been used for surface material classification using an accelerometer and visual information when a rigid tool slides on a surface^46^. Deep-learning methods such as the convolutional neural network and recurrent neural network (RNN) are useful for increasing the classification accuracy^47^. In this research, we demonstrated texture sensing and classification using the Mayan-pyramid sensor with the help of deep learning. From current data captured as the sensor slid across the texture, a LSTM (long-short time memory)^48,49^ network, which is an artificial neural network that recognize patterns in data in the form of time series data, was used for texture classification without a frequency domain analysis. Figure 5 shows surface SEM images of the materials (rough sandpaper, fabric, rough side of a corrugated board, smooth side of a corrugated board, bark, leaf, smooth sandpaper, glass, paper) used here for texture classification in a tactile sensing test. Figure 6 shows the electric current when rubbing a texture with the developed pressure sensor as well as the structure of the proposed network for texture classification.

**Figure 7:**
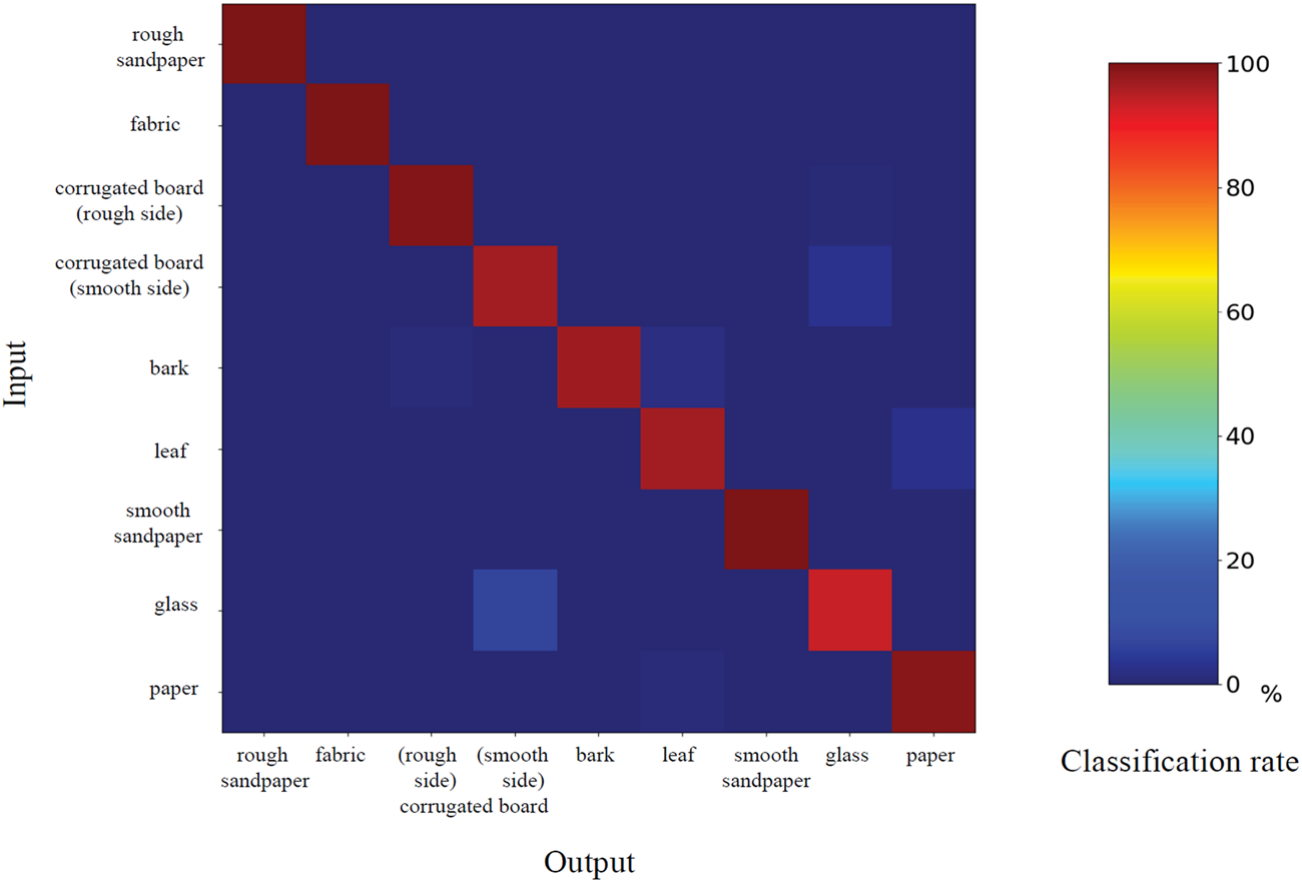
Confusion matrix of the texture classification results.

Table 1 summarizes the detailed architecture of the proposed network. The network inputs are the electrical information, which is given as the input to the LSTM structure using a total of ten instances of sequentially sampled data. Texture classification outcomes are obtained using the output of two layer-based LSTMs as the input of the FC (fully connected) layer and SoftMax^47^. The total number of collected signals is 32544 for each sample for training and for the test. We utilized ten-fold cross validation with the collected database. The final output is measured by averaging the outputs from the ten-fold cross-validation set. Figure 7 shows a confusion matrix of the texture classification outcome using architecture in which the recognition results are in general good, as indicated by the clear diagonal. An overall classification accuracy rate of 98.1% is achieved when the sensor is rubbed on nine different textures. In contrast to previous research, by sensing only tactile information without using visual information, it was possible to classify textures easily and with only a light computational burden due to the small network size. When a tactile sensor is used for robot interaction, it is expected that a plurality of tactile sensors can be attached to various places and that the sensing environment will be similar to human tactile sensing with a light computational burden.

**Table 1:** Proposed Network Configuration.

Texture Classification Analysis	
Layer name	Layer description
Input	9 Mini-batch, 10 Sequence, 1 Channel values
RNN (LSTM Layer 1)	Input = 1, Output = 256, Activation = tanh
RNN (LSTM Layer 2)	Input = 256, Output = 256, Activation = tanh, Dropout = 0.7
FC layer 1	Input = 512, Output = 512, Activation = Leaky_relu (alpha 0.2), Dropout = 0.7
FC layer 2	Input = 512, Output = 1024, Activation = Leaky_relu (alpha 0.2)
Softmax	Input = 1024, Output = 9

### ITO temperature sensor

**Figure 8:**
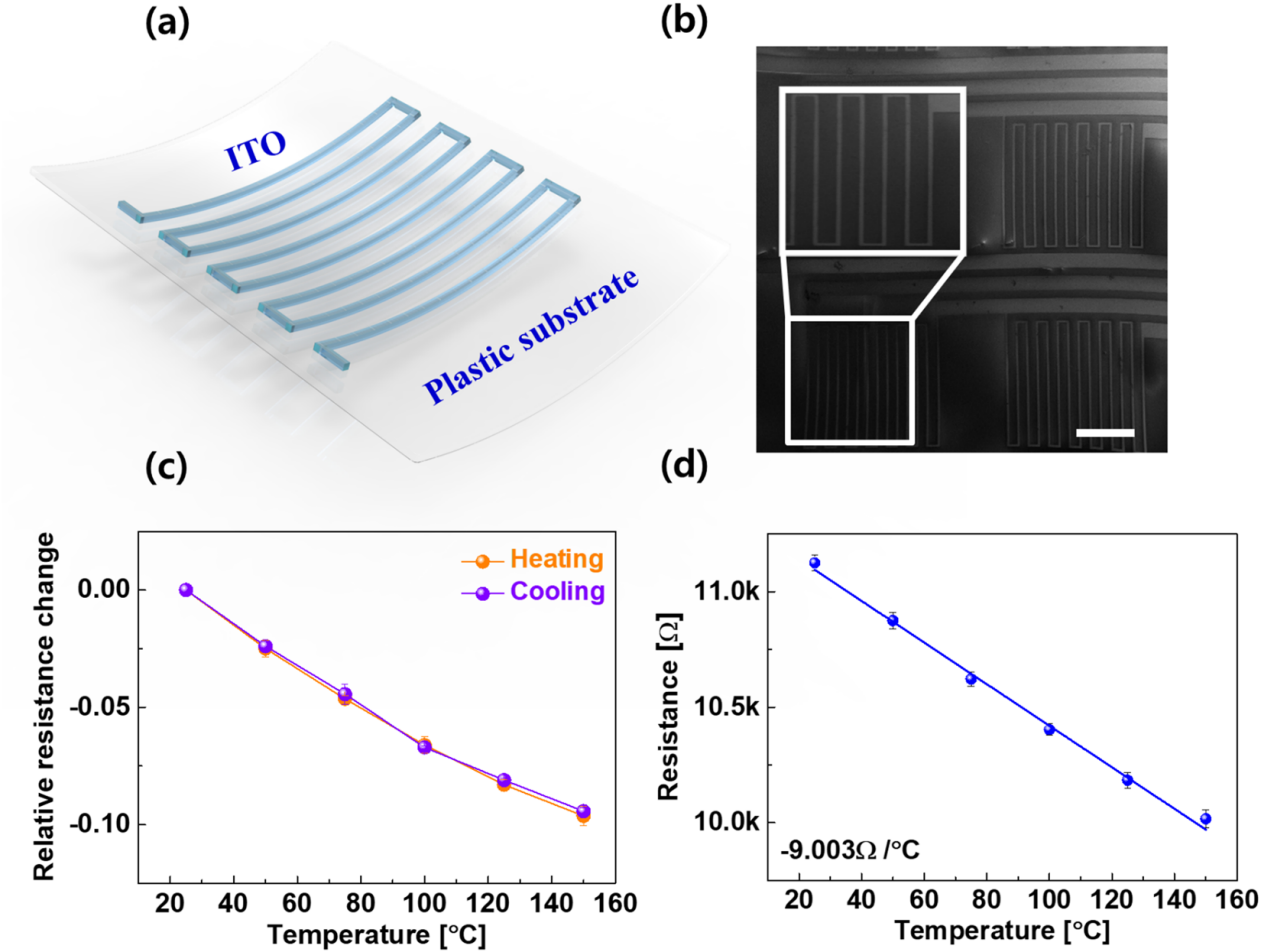
Electrical characteristics of a transparent temperature sensor: (**a**) schematic of the temperature sensor and (**b**) SEM image of the temperature sensor array. The inset shows a single temperature sensor. The scale bar is 2.5 mm. (**c**) Electrical hysteresis upon heating and cooling on the relative resistance change of ITO, and (**d**) negative temperature coefficient of the resistance (NTCR) with ITO for the temperature sensor.

To avoid the requirement of different materials to mimic a transparent bimodal sensor, we utilized ITO as a temperature sensor with a zigzag shape using lithography. A schematic view of the ITO temperature sensor is shown in Fig. 8a, and the SEM microstructure is depicted in Fig. 8b. Similar to many semiconductors, ITO also shows a negative temperature coefficient (NTC) due to the availability of more free carriers upon an increase of the temperature^50–52^. The temperature dependence of the relative resistance change for the zigzag-shaped ITO is shown in Fig. 8c, indicating identical and negative resistance dependence on the temperature (dρ/dT < 0) regardless of any cooling or heating processes, indicative of semiconductor behavior. The temperature-dependent electrical properties of the zigzag ITO are depicted in Fig. 8d. The sensitivity of the temperature sensor is approximately −9 °C, making it feasible for use as a temperature sensor for transparent bimodal sensors.

### Transparent bimodal sensor

**Figure 9:**
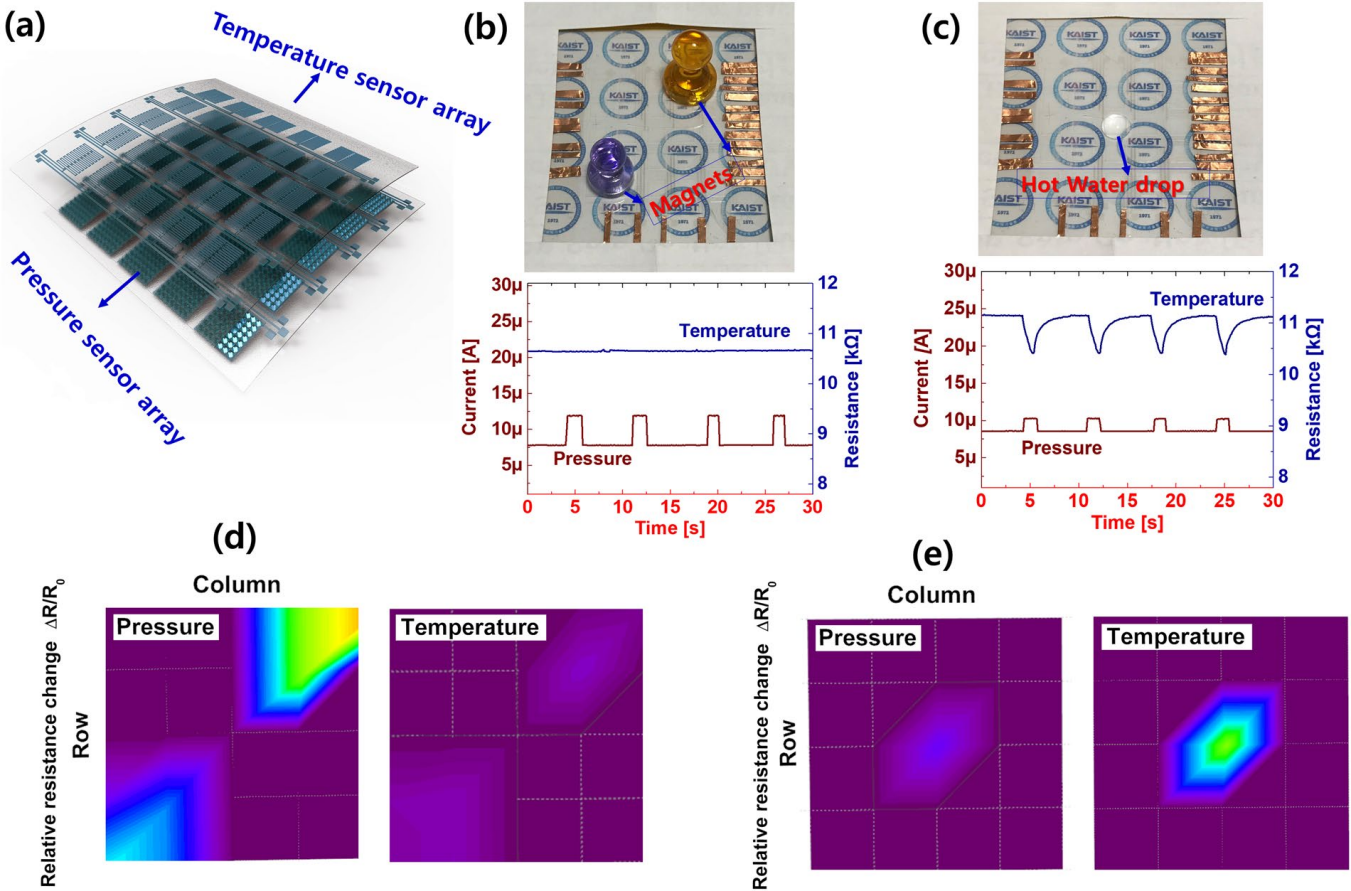
Real time array mapping data of transparent bimodal sensor. (**a**) Schematic view of single pixel of transparent bimodal sensor. Electric measurements for Mutual interference between temperature and pressure sensor when (**b**) small magnets placed on array and (**c**) response for hot water droplet on the array. Real-time mapping array data for (**d**) magnet (**e**) hot water drop.

A schematic view of the transparent bimodal sensor with the 5 × 5 array is shown in Fig. 9a. The electrical interference physiognomies are demonstrated with the help of two small magnets and a hot water droplet. The bimodal sensor only responds to pressure when the magnets are placed on the array due to the negligible temperature gradient between the temperature sensor and the magnets. The corresponding electrical curves are shown in Fig. 9b. When a hot water droplet is dropped onto the sensor array, we observed both the pressure and temperature responses, as shown in Fig. 9c. We also undertook real-time mapping from both the magnet and the water drop on the bimodal sensor array, as shown in Fig. 9d,e, respectively. The above outcomes suggest that our transparent bimodal sensor is applicable for use in electronic skin, or E-skin, which requires multi-functional sensing.

## Conclusion

In summary, we present an ITO-based transparent bimodal sensor for E-skin applications which relies on a combination of a triple-layer or Mayan-pyramid structure as a pressure sensor and a zigzag-shaped transparent temperature sensor. An environmentally friendly method is adopted to fabricate the flexible ITO Mayan-pyramid-based pressure sensor using water-soluble germanium and NaCl as sacrificial layers. This pressure sensor has a wide range of 100 Pa to 10 K Pa for pressure sensing with a quick response time of 80 ms, making it suitable for human health care applications. Tactile sensing is demonstrated from texture classifications of different materials using the output of two-layer-based long-short time memory (LSTM) as the input of the FC (fully connected) layer and SoftMax machine learning (ML) algorithms. In addition, a 5 × 5 pixel bimodal sensor was verified by vertically staging both the pressure sensor array and temperature sensor array using transparent ITO to produce a transparent bimodal sensor for application to E-skin for processes on a larger scale.

## Methods

### Fabrication of a silicon mold for maya pyramids

First, a four-inch thermally oxidized silicon wafer (SiO_2_, 300 nm/P-type boron-doped silicon) with the <100> crystal structure was cleaned by sonication in acetone, ethyl alcohol, and deionized (D.I.) water for five minutes each. Subsequently, negative photoresist (PR) (DNR-L300-30, Dongjin Semichem Co. Ltd.) was spin-coated onto a Si wafer at 1200 rpm for two minutes. Next, square patterns 500 µm long were patterned with photolithography. To etch the silicon oxide (SiO_2_) layer on the silicon wafer, it was wet etched in buffered oxide etchant (BOE). The wafer was then cleaned to remove the remaining PR, after which 44:56 wt% of potassium hydroxide (KOH) in the form of an aqueous solution was used to etch the silicon wafer. Due to the natural characteristics of <100> crystalline silicon, it was etched with a negative pyramid structure at 90 degrees with the KOH solution for two hours. Before being fully etched in the form of a pointed pyramid, the wafer was washed with D.I. water and a SiO_2_ layer 300 nm thick was deposited with a furnace in a water vapor ambient condition. Lastly, we repeated the previous steps two times to fabricate square patterns with dimensions of 300 µm by 100 µm (length). As the size of the square pattern for the formation of the pyramid structure became smaller, a pyramid mold with a multilayer structure could be manufactured.

### Fabrication of the bimodal sensor

500 nm of NaCl and 200 nm of Ge were deposited on the Mayan-pyramid-structured silicon mold with a thermal evaporator as a sacrificial layer which could be dissolved in water. Next, an indium tin oxide (ITO) conductive layer was deposited at 200 nm. Subsequently, PDMS was poured onto the silicon mold. To cure the PDMS, we used an oven at 60 °C for 30 minutes, and in order to detach the PDMS from the silicon mold, we used hot water as an etching solution for the NaCl/Ge sacrificial layer. After 15 mins of etching, the sacrificial layer was fully removed and the ITO-embedded PDMS was detached from the prepared pyramidal-structured Si-mold. This ITO-embedded PDMS in the form of a pyramid structure will serve as the bottom electrode of the pressure sensor in the bimodal sensor. For the counter electrode of the pressure sensor, we used ITO-deposited PET. At the same time, on the backside of the counter electrode, we fabricated an ITO temperature sensor with a zigzag pattern through photolithography.

### Characterization

The electrical characteristics of the transparent bimodal sensor were assessed using a universal measured probe system (Tera Leader UMP-1000). The measurement of the pressure sensor was evaluated using a custom-built pressure-application system. While a wide range of pressures was applied, from 500 Pa to 11 kPa, the electrical characteristics were assessed with a semiconductor parameter analyzer (Agilent 4156 C). At the same time, a parameter analyzer (Keithley 4200A-SCS) was used to measure the resistance change of the ITO temperature sensor. Because the electrical signals of the pressure sensor and the temperature sensor are separate from each other, we could ignore the possibility of mutual interference effects. Surface profiles for a detailed material analysis of the Mayan-pyramid structure and the zigzag temperature sensor pattern were observed by field-emission scanning electron microscopy (Tescan MIRA3).

## Supplementary information

Supporting Information
