# Supplementary material for: Transparent and Flexible Mayan-Pyramid-based Pressure Sensor using Facile-Transferred Indium tin Oxide for Bimodal Sensor Applications
Source: Sci Rep. 2019 Oct 1;9:14040. doi: 10.1038/s41598-019-50247-4 (PMC6773852; doi:10.1038/s41598-019-50247-4)
Supplement: Supplementary file 1 — Supporting Information [file 41598_2019_50247_MOESM1_ESM.docx]

**Transparent and Flexible Mayan-Pyramid-based Pressure Sensor using Facile-Transferred Indium tin Oxide for Bimodal Sensor Applications**

*Minhyun Jung^1, †^, Sujaya Kumar Vishwanath^1, †^, Jihoon Kim^2^, Dae-Kwan Ko^3^, Myung-Jin Park^3^, Soo-Chul Lim^3^*, and Sanghun Jeon^1^ **

^1^Korea Advanced Institute of Science and Technology (KAIST), School of Electrical Engineering, Daejeon, 34141, Republic of Korea.

^2^Division of Advanced Materials Engineering, Kongju National University, Cheonan, Chungchungnam-do 331-717, Republic of Korea.

^3^Department of Mechanical, Robotics and Energy Engineering, Dongguk University, Seoul, 04620, Republic of Korea

*Corresponding author: [jeonsh@kaist.ac.kr](mailto:jeonsh@kaist.ac.kr), limsc@dongguk.edu

^†^Authors contributed equally


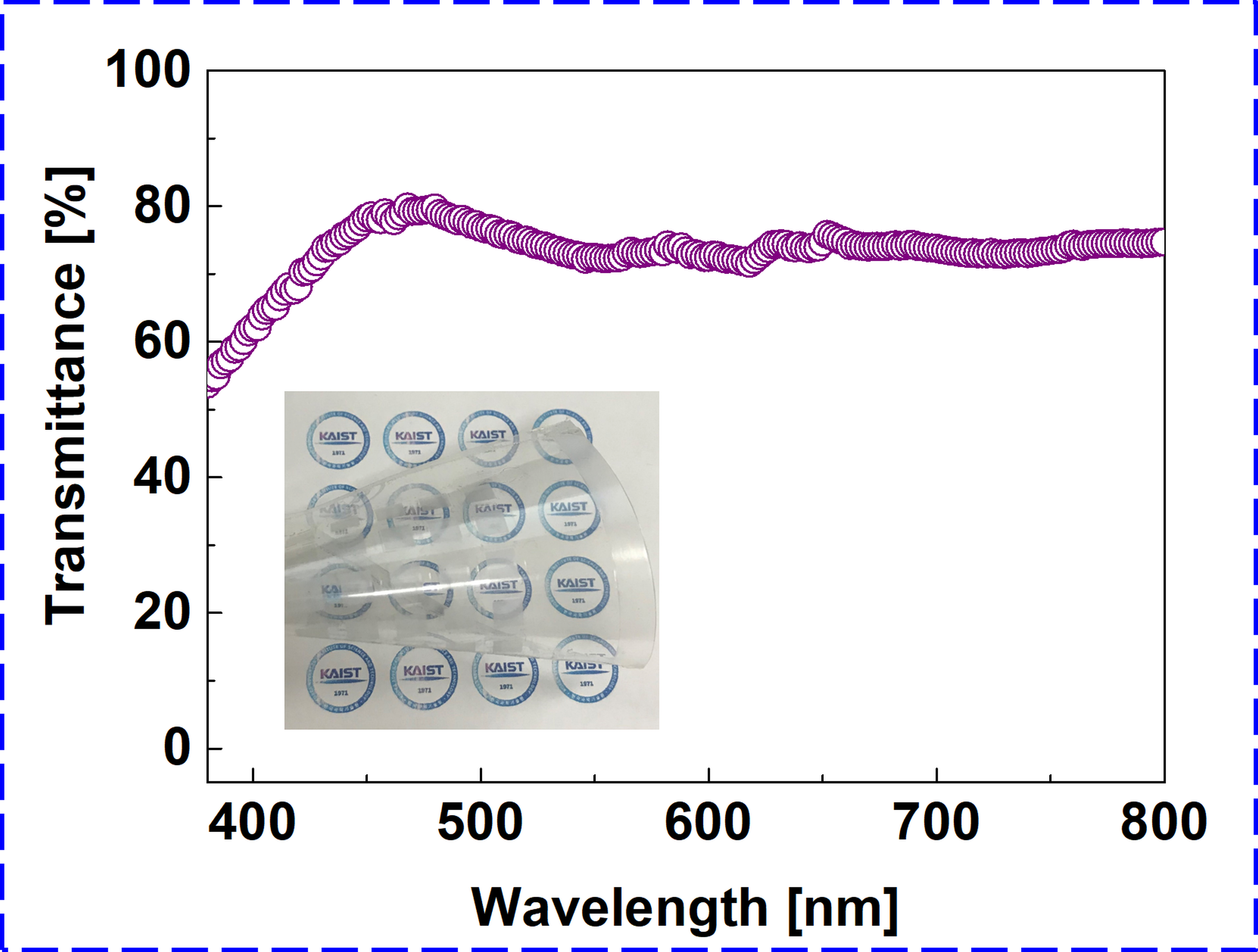


**Figure S1..** (a) Optical Transmittance of triple layer prymaind ITO in PDMS (inset: Optical image) .


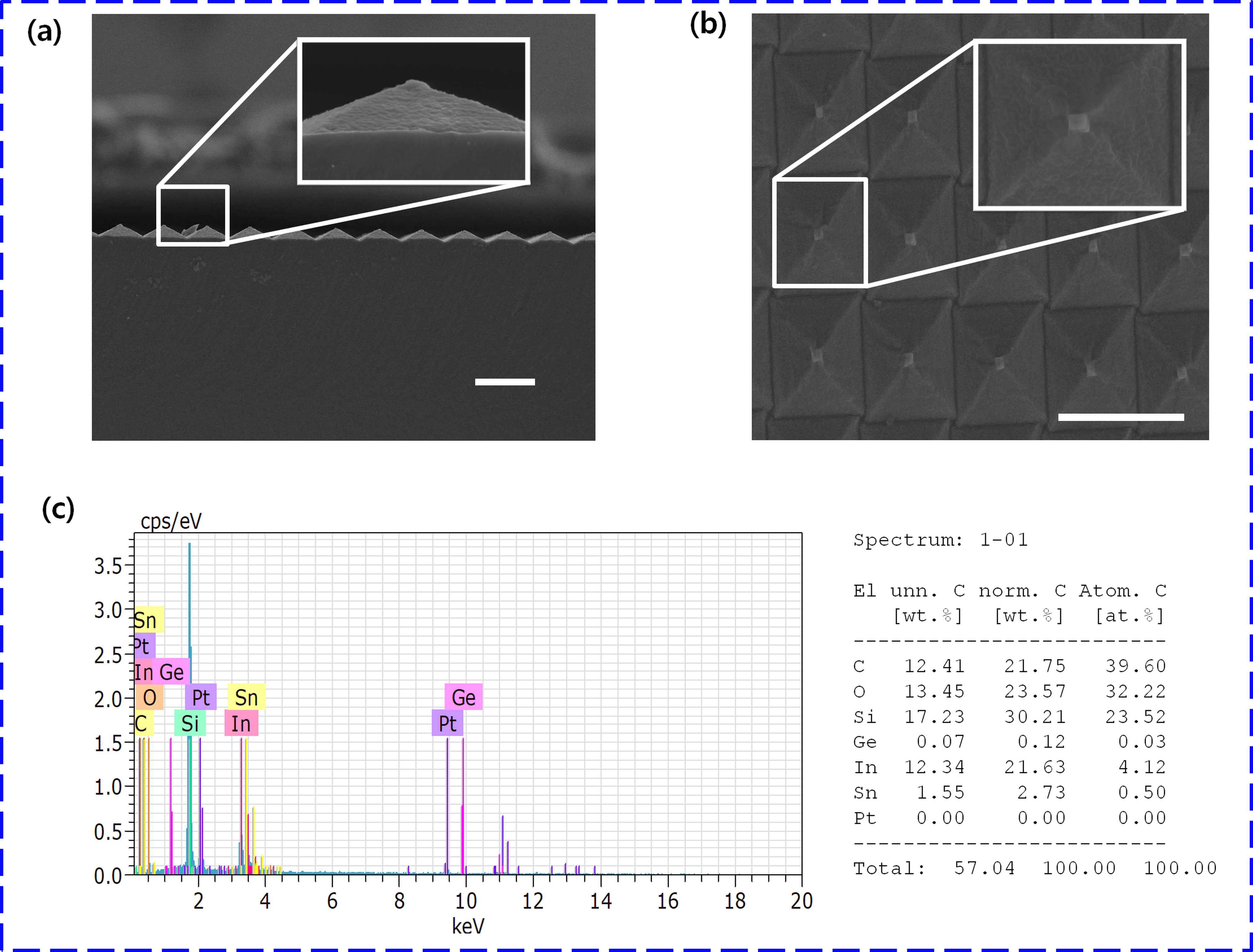


**Figure S2. Material analysis of conductive pyramid layer after water soluble process.** (a) Plane view SEM image of pyramid structure and (b) vertical view. Scale bar is 100um. (c) Energy dispersive spectroscopy (EDX) data shows there are no remaining Ge/NaCl component after etching process with hot water.
